# Root Structural and Metabolic Plasticity Confers Tolerance to Salinity in Wild Barley Species Grown Under Waterlogging

**DOI:** 10.1111/pce.70563

**Published:** 2026-04-23

**Authors:** Stanislav Isayenkov, Ljudmilla Borisjuk, Simon Mayer, Alexander Hilo, Dominic Knoch, Tobias Meitzel, Bettina Hause, Yudelsy Antonia Tandron Moya, Hardy Rolletschek, Edgar Peiter, Volodymyr Radchuk

**Affiliations:** ^1^ Leibniz‐Institute of Plant Genetics and Crop Plant Research (IPK), Corrensstrasse 3 Germany; ^2^ Institute of Agricultural and Nutritional Sciences Martin Luther University Halle‐Wittenberg Halle Germany; ^3^ Department of Plant Food Products and Biofortification Institute of Food Biotechnology and Genomics, National Academy of Sciences of Ukraine Kyiv Ukraine; ^4^ Department of Cell and Metabolic Biology Leibniz Institute of Plant Biochemistry Halle Germany

**Keywords:** root anatomical plasticity, salinity stress, stress metabolic adjustments, tissue‐specific ion distribution, waterlogging, wild barley relatives

## Abstract

Salinity combined with waterlogging is a major abiotic stress that severely limits crop growth and yield. We investigated species‐specific adaptations to salinity under constant waterlogging conditions in the wild halophytic barleys *Hordeum marinum* and *H. glaucum*, compared with the cultivated *H. vulgare*. Using magnetic resonance imaging, fluorescence scanning microscopy, ¹³C‐based carbon fixation analysis, and ion and metabolite profiling, we identified key anatomical and physiological traits underlying differential salinity responses. *H. marinum* exhibited the highest tolerance under saline waterlogging, maintaining water status, metabolic activity, and high carbon fixation rates. This species accumulated the lowest concentrations of Na⁺ and Cl⁻ while retaining the highest levels of K⁺ in both roots and shoots. *H. glaucum* showed intermediate tolerance associated with reduced water content, whereas *H. vulgare* failed to survive under these conditions. We propose that salinity tolerance in *H. marinum* is mediated by an integrated root‐based mechanism in which intact aerenchyma sustains internal oxygen transport. At the same time, salt‐induced enhancement of lateral root branching promotes sequestration of excess Na⁺ within the lateral root cortex, thereby limiting its translocation to photosynthetically active tissues. This aeration–sequestration system stabilizes root function under salinity and waterlogging, and promotes whole‐plant resilience in wild barleys, but is only weakly maintained in cultivated *H. vulgare*.

## Summary statement

Wild barley species show enhanced tolerance to saline waterlogging through root anatomical plasticity, Na⁺ accumulation in newly branched roots, and maintained metabolic activity, with *Hordeum marinum* displaying the strongest ability to restrict Na⁺ transport to shoots and sustain carbon fixation.

## 1 Introduction

Salinity and drought are major threats to modern agriculture, significantly reducing global crop productivity. High soil salinity is particularly prevalent in arid, semi‐arid, and coastal regions. Furthermore, large areas of agricultural land are waterlogged, often accompanied by salinity (Barrett‐Lennard 2003). Worldwide, nearly 20% of irrigated land is classified as salt‐affected, leading to substantial crop yield reductions, sometimes resulting in complete yield loss (Food and Agriculture Organization 2016). With ongoing climate change, rising sea levels, and decreasing water availability for agriculture, these challenges will become increasingly difficult to address.

Sodium (Na^+^) and chloride (Cl^–^) are the most abundant ions in saline soils and have the most harmful effects on plant growth and development. They negatively impact the uptake of essential nutrients such as K^+^ and Ca^2+^, reduce water absorption, alter metabolism, cause ionic imbalances, induce toxicity (Munns and Gilliham 2015), and affect many other biosynthetic processes, including photosynthesis (Lindermayr and Durner 2015). Many plant species have developed unique strategies to survive and thrive in harsh saline environments.

In addition to salinity stress, plants are frequently exposed to waterlogging, particularly in coastal environments and flooded soils, which results in hypoxic conditions that impair mitochondrial adenosine triphosphate (ATP) production. This reduced energy availability compromises nutrient uptake and carbon assimilation, thereby limiting growth and development (Palmgren and Shabala 2024). With the exception of rice, most staple crops are highly sensitive to hypoxia in the rhizosphere caused by waterlogging, and yield losses in susceptible species can reach up to 70% (Liu et al. 2020; Rolletschek et al. 2024; Sharmita et al. 2026). To cope with hypoxia in the submerged roots, some plants, including barley, have evolved a range of adaptive traits related to oxygen supply, including the formation of adventitious and lateral roots, the establishment of radial oxygen loss barriers through suberin and lignin deposition in outer root tissues to reduce oxygen leakage, and the development of aerenchyma to enhance internal oxygen diffusion (Evans 2004; Yamauchi et al. 2018; Zhang et al. 2017; Manik et al. 2022a, 2022b; Herzog et al. 2023).

Within the Triticeae tribe, which includes such globally important cereal crops as wheat and barley, particular attention deserves their wild relatives that exhibit halophytic phenotypes while retaining similar anatomy, physiology, and genome structure. Among these, Hordeum I‐genome species have gained increasing relevance due to their exceptional stress tolerance and genetic diversity (Feng et al. 2025). Tolerance‐associated genes from wild barley relatives are considered more amenable to introgression into modern cereals or targeted modification to meet agricultural demands (Isayenkov 2019). Although the genus Hordeum comprises numerous wild species exhibiting superior tolerance to salinity, drought, and waterlogging, only a limited number of studies have systematically explored the mechanisms of their stress tolerance (Garthwaite et al. 2005; Isayenkov 2019, 2023).

*H. marinum*, native to coastal regions and salt marshes, is capable of growing and reproducing at salinity levels of up to 1.8% and is therefore considered a key donor of salt‐tolerance genes for other cereals, including wheat (Colmer et al. 2006; Islam et al. 2007; Kuang et al. 2022; Xu et al. 2025). The development *of H. marinum*–*Triticum aestivum* amphiploids has resulted in plants with intermediate salt and waterlogging tolerance compared with their parental species (Islam et al. 2007; Alamri et al. 2013). *H. marinum* exhibits a remarkable capacity to form a barrier that reduces radial oxygen loss from roots, making it a valuable model for studying waterlogging tolerance (Malik et al. 2009; Alamri et al. 2013; Konnerup et al. 2017; Xu et al. 2022). *H. marinum* forms constitutive aerenchyma and displays high gas‐filled porosity in roots, both of which can further increase under waterlogging and hypoxic conditions in the root zone (Garthwaite et al. 2003). In contrast, the roots of domesticated wheat and barley, typical dryland crops, do not form constitutive aerenchyma and produce only limited inducible aerenchyma in response to waterlogging (Luan et al. 2018; Gao et al. 2025; Jin et al. 2025). Metabolic adjustments to waterlogging and salinity stress in *H. marinum* include pronounced changes in energy metabolism, as well as the accumulation of osmoprotective compounds such as proline, dehydrins, amino acids, and sugars (Maršálová et al. 2016). The salt‐tolerance strategy of *H. marinum* further includes up‐regulation of glycolysis and the tricarboxylic acid (TCA) cycle to sustain energy supply to the shoot, utilization of inorganic ions as energetically efficient osmolytes, and modifications in the activity of membrane transporters involved in Na^+^, K^+,^ and Cl^–^ transport (Huang et al. 2018; Isayenkov et al. 2020; Kuang et al. 2022; Chen et al. 2025). Collectively, these studies indicate that *H. marinum* fine‐tunes membrane transport to regulate ion uptake and sequestration across different organs, enhances osmolyte accumulation, and minimizes energy costs under stress conditions. To gain deeper insight into the adaptive mechanisms underlying salinity and waterlogging tolerance, it is therefore essential to characterize the tissue‐specific distribution patterns of Na⁺, Cl⁻, and K⁺ ions in both roots and shoots (Kotula et al. 2015).

Several other wild barley species exhibit superior salt tolerance. We have recently collected *H. glaucum* (syn. *H. murinum* subsp. *glaucum* (Steud) Tzvelev) plants from the salt flats of Larnaca Salt Lake, Cyprus. This wild barley species was found growing alongside canonical halophytes such as *Salicornia* sp. (Patel 2016). However, most of the recent studies have focused on single‐species analyses or specific physiological processes, limiting a systems‐level understanding. To address this gap, in the present study, we have applied an integrated, multi‐method comparative approach to examine adaptation strategies to high salinity across three barley species with contrasting tolerance levels: the cultivated *Hordeum vulgare*, the moderately salt‐tolerant wild relative *H. glaucum*, and the halophytic *H. marinum*. Our findings support the hypothesis that root anatomical plasticity and metabolic adjustments play a critical role in maintaining ion and water homoeostasis at the whole‐plant level under salt stress and waterlogging, thereby conferring high salinity tolerance in the halophytic *H. marinum* and intermediate salt tolerance in the wild relative *H. glaucum* compared with cultivated *H. vulgare*.

## 2 Materials and Methods

### 2.1 Plant Material and Growth Conditions

Seeds of *Hordeum marinum* (syn. *H. marinum* subsp. *marinum* Huds) from the Tuscany region (Italy), *H. glaucum* (syn. *H. murinum* subsp. g*laucum* (Steud) Tzvelev) from the shore of Larnaca Salt Lake (Cyprus), and cultivated barley (*H. vulgare* L.) cv. Golden Promise, received from Genbank IPK Gatersleben, was germinated on moist filter paper in the dark at 20°C. Seven‐day‐old seedlings were transferred to a hydroponic system with half‐strength Hoagland's No. 2 solution (Sigma‐Aldrich, St. Louis, MO, USA) and incubated in a growth chamber under an irradiance of 350 µmol m^−2^ s^−1^, with a 12 h photoperiod, at 20°C under stagnant (no additional solution aeration) conditions. The incubation solution was completely replaced weekly to prevent nutrient depletion. After 14 days of growth, plants were subjected to increasing concentrations of 50 mM NaCl daily until a concentration of 300 mM NaCl was reached. All plants were sampled after 8 days of maximum stress (35 days old) and separated into the shoot (crown and growing point) and root fractions for further analyses. To average the genetic background and local environmental influences, a bulk of 7–10 plants, grown in a single hydroponic tank under either control or salt‐stress conditions, was collected for one biological replication per barley species. At least three independent biological replicates, each derived from separate tanks, were analysed.

### 2.2 Determination of Physiological Characteristics

The relative growth rate (RGR) was calculated from the fresh weight data taken at the start of stress application and final harvest using the formula 1:

(1) $$\mathrm{RGR}=\frac{\ln\left({\text{FW}}_{2}\right)\;-\;\ln\left({\text{FW}}_{1}\right)}{t_{2}-t_{1}}$$

where FW_1_ and FW_2_ represent the fresh weights (g) at the start (*t*_1_) and end (*t*_2_) of the experiment, with time measured in days. The RGRs of individual plants were presented as percentages relative to the value in control conditions.

Fresh plant tissues were dissected and collected in 1.5 mL Eppendorf microcentrifuge tubes for further metabolic and ionomic analyses and stored at −80^°^C. The tissue sap of separated roots and shoots was extracted by squeezing fresh biomass with a Pellet pestle (Eppendorf, Germany). The osmotic pressure of experimental solutions and sap obtained from the squeezed root and shoot tissues was measured using a WESCOR 5500 vapour pressure osmometer (Thermo Fisher Scientific, Germany) according to the manufacturer's instructions. Measurements were conducted for the three individual plants of each species and treatment.

The fresh weight (FW) of individual *H. vulgare, H. glaucum,* and *H. marinum* plants was measured using a standard lab analytical balance. The dry weight (DW) of the plants was determined by drying in an oven at 70°C until they reached a constant weight. Plant water content (PWC) was estimated using formula 2:

(2) $$\mathrm{PWC}=\frac{\mathrm{FW}-\mathrm{DW}}{\mathrm{DW}}$$

The PWC values were converted into percentages relative to the value in the control conditions. Statistical significances between control and stress treatments were assessed using Student's *t*‐test.

### 2.3 Tissue Mineral Content Analyses

~10 mg of pulverized and dried at 65°C plant material was weighed into PTFE digestion tubes, and 1 mL of concentrated nitric acid (67%–69%) was added to each tube. After 4 h incubation, samples were digested under pressure using a high‐performance microwave reactor, Ultraclave 4 (MLS, Germany). Samples were then transferred to Greiner centrifuge tubes and diluted with de‐ionized water to a final volume of 8 mL. Elemental analysis was performed using a sector field high‐resolution mass spectrometer (HR)‐ICP‐MS ELEMENT 2 (Thermo Fisher Scientific, Germany) with software version 3.1.2.242. A 10‐point external standard calibration curve was prepared from a certified multiple standards solution (Bernd Kraft, Germany). A least‐squares regression was applied to best fit the linearity of the curve. Elements Rh and Ge (ICP Standard Certipur®, Merck, Germany) were infused online and used as internal standards for matrix correction.

To analyse mineral content in the different root zones, ~20‐mm‐long root segments were collected from the root tip region, the mature root zone, and newly formed lateral and adventitious roots (10 mm below the crown). The experiment was conducted four times, and the statistical significance among root zones was assessed using one‐way ANOVA followed by Tukey's honest significant difference (HSD) test.

### 2.4 Magnetic Resonance Imaging of Plant Tissues

The internal tissue structure and water distribution in roots of stressed and control plants were investigated non‐invasively on a Bruker Avance III HD 400 MHz NMR spectrometer (Bruker BioSpin, Germany) equipped with a 25 mm inner‐diameter ^1^H quadrature RF resonator as established earlier (Munz et al. 2017). Three‐dimensional T₁‐weighted spin‐echo images were acquired with TR = 500 ms, TE = 8.3 ms, FOV = 20 × 14 × 14 mm³, and an isotropic resolution of 50 µm. For proton density‐weighted imaging, a multi‐slice spin‐echo sequence (30 slices) was used with TR = 6 s, TE = 5.6 ms, slice thickness = 0.2 mm, interslice ga*p* = 0.7 mm, FOV = 17 × 17 mm², and resolution = 60 µm. Eight averages were acquired to improve the signal‐to‐noise ratio. An NMR tube filled with water served as a reference; image intensities were normalized to this phantom to enable comparison across experiments. Image processing was performed by application of software MATLAB (The Mathworks, Natick, MA, USA) and AMIRA (Thermo Fisher Scientific, Germany).

### 2.5 Measurement of ^13^C Uptake

Bag‐covered hydroponic tanks containing plants grown under control or stressed conditions were exposed to 400 ppm ^13^CO_2_ for 24 h. Afterwards, treated plants were separated into shoot and root fractions, lyophilized, powdered, and analysed on an elemental analyser coupled to a stable isotope ratio mass spectrometer Vario MICRO cube/Isoprime Vision (Elementar Analysensysteme, Germany). Five biological repetitions with three technical replicates each were analysed. Statistical significance between control and stress plants within a species was assessed using Student's t‐test.

### 2.6 Untargeted Metabolite Profiling

For the untargeted analysis of central metabolites, the freeze‐dried and homogenized samples were prepared and measured essentially as described earlier (Isayenkov et al. 2020) with the same chromatographic and mass spectrometry (MS) conditions. The randomized samples were analysed in full MS mode. The data‐dependent MS‐MS analysis for the compound identification was performed in the pooled probe, which also served as a quality control (QC). The batch data was processed using the untargeted metabolomics workflow of the Compound Discoverer 3.0 software (Thermo Fisher Scientific, Germany). The compounds with the maximum relative standard deviation (RSD) below 35% of the QC area were selected for quantification. Compounds were identified using an in‐house library, as well as the public spectral database mzCloud and the public databases KEGG, NIST, and ChEBI via the mass‐ or formula‐based search algorithm. Adjusted *p*‐values accounting for multiple testing were calculated using Benjamini–Hochberg correction method.

### 2.7 Confocal Laser Scanning Microscopy

Na^+^ and K^+^ distribution in barley root tissues was visualized using CoroNa Green AM (Invitrogen, Thermo Fisher Scientific, US) and ION Potassium Green‐2 AM (Abcam, UK) dyes, respectively, following Wu et al. (2015) and Lee et al. (2022). Root segments of control and stressed plants were collected from the root tips (10 mm from the apex), mature zone (20–30 mm from the apex), and upper newly formed lateral and adventitious roots (10–15 mm below the crown). Samples were embedded in 5% agarose buffered with 10 mM KCl or NaCl and 5 mM Ca^2+^‐MES (pH 6.1) for Na^+^ and K^+^ labelling, respectively, and sectioned into 100 µm sections by a vibratome (Leica, Germany). To ensure full penetration of the ion‐specific dyes through the dense cell walls and membranes of plant tissues embedded in agarose, the sections were incubated overnight at 4°C in the dark in buffer containing either 40 µM CoroNa Green AM or ION Potassium Green‐2 AM, washed twice with the same buffer, and immediately imaged. Fluorescence was detected using a Zeiss LSM900 confocal microscope (Carl Zeiss, Germany) using a 40× oil immersion objective (filter set Ex/Em = 488/516 nm for CoroNa Green AM; Ex/Em = 526/546 nm for ION Potassium Green‐2 AM). All images were acquired using identical microscope settings (i.e., exposure time, laser intensity, pinhole diameter, and settings of the imaging detectors). Ion distribution was analysed from three to five randomly selected sections per root zone, obtained from four independent plants.

## 3 Results

### 3.1 Physiological and Anatomical Responses of *H. marinum, H. glaucum and H. vulgare* to Salinity

**Figure 1:**
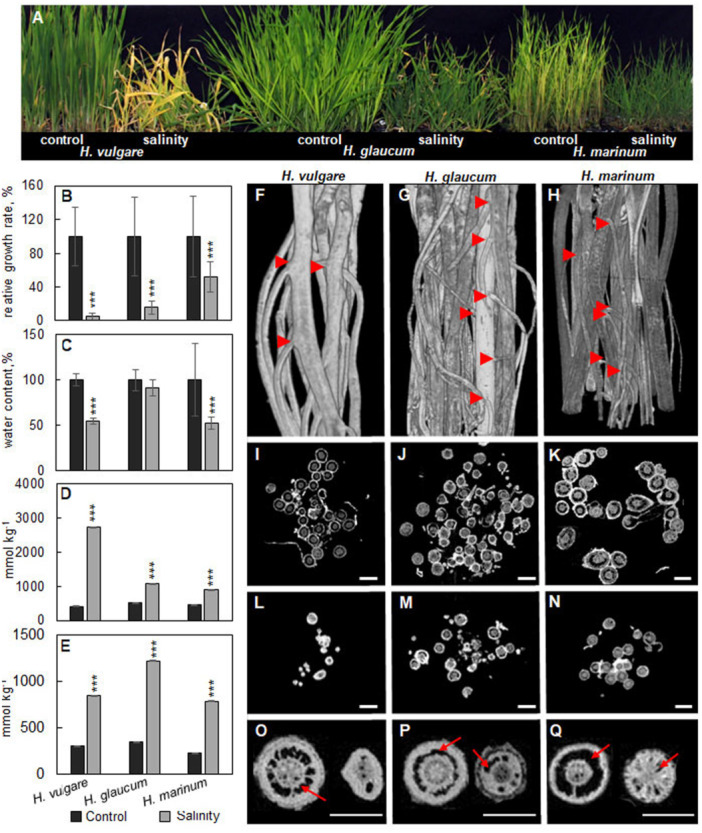
Phenotypic appearance of *Hordeum vulgare*, *H. glaucum,* and *H. marinum* plants grown under control hydroponic and salinity stress conditions. (A) Shoot phenotype; (B) Relative growth rate; (C) Plant water content; (D, E) Osmotic pressure of tissue sap from shoots (D) and roots (E). Data are mean ± SD; *n* = 5, *t* significant (Student's *t*‐test) at: ***p < 0.001. (F–Q) Root phenotypes as analysed by Magnetic Resonance Imaging. (F, G) 2D projections of 3D images of salt‐stressed roots of *H. vulgare* (F), *H. glaucum* (G), and *H. marinum* (H). The 3D images are shown in the Supplementary information Movies S1 and S2. Red triangles point to root branches. (I–N) Virtual cross sections of control (I–K) and salt‐stressed roots (L–N) of *H. vulgare* (I, L), *H. glaucum* (J, M), and *H. marinum* (K, N). (O–Q) Virtual cross sections of individual control (left) and salt‐stressed roots (right) of *H. vulgare* (O), *H. glaucum* (P), and *H. marinum* (Q). Bars, 1 mm in (I–N), 500 µm in (O–Q).

To analyse species‐specific responses to salinity, young *H. vulgare*, *H. glaucum,* and *H. marinum* plants were cultivated in a hydroponic system under control and salinity conditions. Analysis of whole‐plant fresh and dry biomass revealed that salinity inhibited growth in all three species, albeit to markedly different extents. *H. marinum* plants continued to grow under saline conditions, although their growth rate was reduced by 48% compared to the control conditions, whereas *H. glaucum* exhibited a pronounced growth reduction of approximately 85% (Figure 1A,B). In contrast, salinity treatment in *H. vulgare* resulted in an almost complete cessation of growth, corresponding to a 96% reduction.

The ability to retain water in tissues under saline conditions, relative to the control, also differed among species. *H. marinum* retained 65% of its tissue water content, *H*. *glaucum* 92%, whereas H. vulgare retained only 54% (Figure 1C). Correspondingly, a significant increase in osmotic pressure was observed in all three barley species (Figure 1D,E). Both *H. marinum* and *H. glaucum* maintained significantly lower osmotic pressure in their shoots than salt‐treated *H*. *vulgare*. Whereas osmotic pressure in the roots of all species increased by approximately threefold under salinity, shoot osmotic pressure increased by only about twofold in salt‐treated *H. marinum* and *H. glaucum*. In contrast, osmotic pressure in the shoots of salt‐treated H. vulgare increased by more than sixfold.

Three‐dimensional (3D) visualization of the root systems of *H. vulgare*, *H. glaucum,* and *H. marinum* plants by Magnetic Resonance Imaging (MRI) enabled a comparative analysis of root architecture under control and salinity conditions (Figure 1F,G, Movies [Link], [Link]). Under control conditions, all three species developed branched roots with pronounced aerenchyma (Figure 3O–Q, Movies S1), likely reflecting plant adaptive responses to hydroponic growth. Aerenchyma generally facilitates tolerance to waterlogging by providing internal air spaces that support gas exchange (Zhang et al. 2025). Under saline conditions, *H. glaucum* and *H. marinum* maintained root branching and produced a high proportion of fine lateral and adventitious roots, resulting in the formation of a well‐developed fibrous root system (Figure 1F–H, Movie S2). However, the aerenchyma regions were less prominent in the salt‐stressed roots (Figure 1P,Q). In contrast, *H. vulgare* ceased the production of new roots under salinity stress and exhibited pronounced root degradation manifested by root shrinkage and loss of internal tissue organization (Figure 1F,L,O).

### 3.2 Salinity Stress Drives Changes in Root Water Distribution

**Figure 2:**
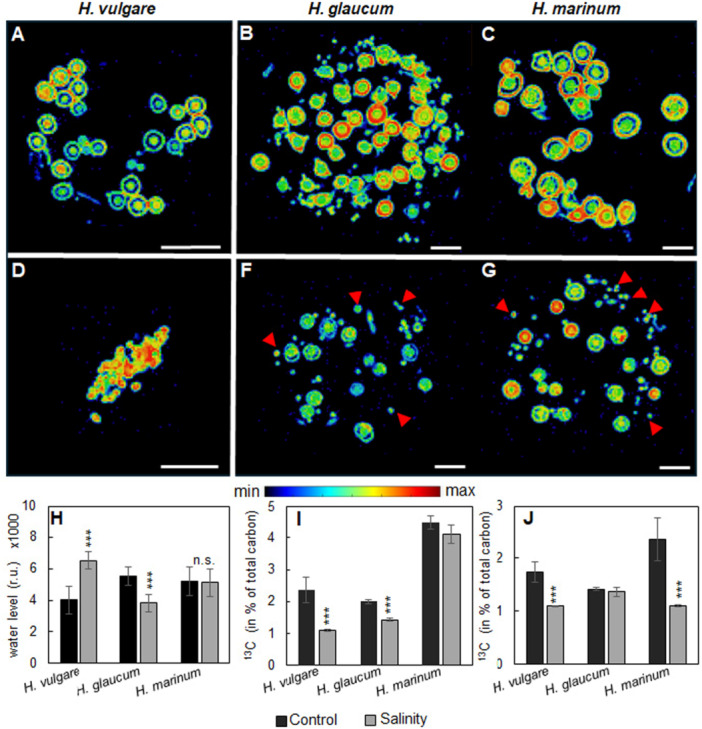
Root water distribution, and ^13^C‐uptake and distribution in plants under hydroponic control and salt‐stressed conditions. (A–G) Water distribution in virtual cross sections of control (A–C) and salt‐stressed roots (D–G) of *H. vulgare* (A, D), *H. glaucum* (B, F), and *H. marinum* (C, G) as analysed by Magnetic Resonance Imaging. Min/max values of the colour bar represent the relative water concentrations. Red triangles indicate new formed roots under salinity stress. Bars, 1 mm in (A, D), 500 µm in (B, C, F, G). (H) Quantification of water content in roots of three barley species analysed in (A–G). (I, J) ^13^C‐uptake and distribution in shoots (I) and roots (J) of three barley species under control and salinity stress conditions. Data are mean ± SD; *n* = 7–25 in (H), *n* = 3–5 in (I, J), *t* significant (Student's *t*‐test) at: ***p < 0.001, n.s., not significant. [Color figure can be viewed at wileyonlinelibrary.com]

MRI enables quantitative imaging of water (Borisjuk et al. 2023), which allows us to investigate water distribution in the root and relative water content within the distinct root tissues (Figure 2A–H). Under control conditions, higher water signals were detected in the root cortex than in the stele (containing vascular tissues), with clearly delineated water‐free aerenchyma in all three species (Figure 2A–C). Under salinity stress, *H. vulgare* roots displayed highly irregular water distribution, and the aerenchyma spaces were largely filled with water (Figure 2D), indicating loss of root integrity. Quantitative MRI measurements revealed a significant increase in total water content in *H. vulgare* roots, consistent with extensive root disintegration (Figure 2H). In contrast, roots of *H. glaucum* and *H. marinum*, which had developed before stress application, largely preserved their tissue organization, with still defined aerenchyma. Newly formed, smaller lateral and adventitious roots exhibited reduced aerenchyma volumes (Figure 2K,L). Quantification of MRI results showed a ~30% decline in root water content in *H. glaucum* under salinity stress, while *H. marinum* maintained stable root water levels (Figure 2H).

Taken together, MRI analysis demonstrates that salinity differentially affected root architecture, aerenchyma formation, and water distribution among *Hordeum* species. The non‐halophytic *H. vulgare* underwent severe structural collapse under salt stress, whereas the salt‐tolerant species *H. glaucum* and *H. marinum* formed numerous adventitious roots and largely preserved aerenchyma function in roots developed prior to stress exposure. Notably, newly formed roots of *H. marinum* are characterised by reduced root diameter and aerenchyma spaces. *H. glaucum* had a markedly reduced tissue water content, while *H. marinum* uniquely maintained a near‐constant water level.

### 3.3 Salinity Stress Affects Carbon Fixation and Assimilate Allocation

To analyse photosynthetic carbon fixation and assimilate allocation in *H. marinum*, *H. glaucum,* and *H. vulgare* under saline conditions, ^13^C‐labelled CO₂ was applied to the shoots of control and salt‐stressed plants, and ^13^C accumulation and distribution were subsequently monitored (Figure 2I,J). Under stress conditions, *H. marinum* shoots exhibited approximately twofold higher ^13^C uptake efficiency than those of *H. vulgare* and *H. glaucum*, with little reduction in uptake under salinity (Figure 2I). These results indicate that *H. marinum* is able to maintain efficient photosynthetic activity under saline conditions. Under control conditions, all species allocated substantial amounts of ^13^C‐labelled assimilates to the roots. Notably, salinity stress caused a significant reduction in ^13^C allocation to the roots in *H. vulgare* and *H. marinum*, but not in *H. glaucum* (Figure 2J).

### 3.4 Alterations of Metabolite Profiles in Response to Salinity Stress in Three Barley Species

**Figure 3:**
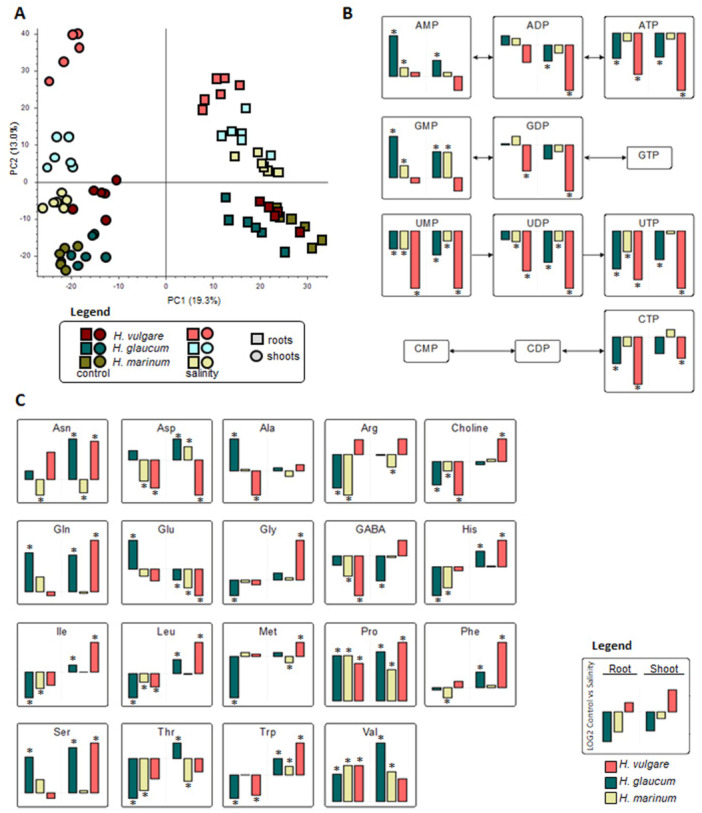
The effect of salinity stress on metabolite content in three barley species. (A) Principal component analysis of metabolite distribution in roots and shoots of *H. vulgare*, *H. glaucum,* and *H. marinum* plants grown under control and salinity stress conditions. (B, C) Changes in nucleotide (B) and amino acid content (C). Metabolomic data in (B, C) are shown as log_2_(fold‐change) values of the relation salinity to control, negative values mean decreased metabolite content, positive values – increased metabolite content. Adjusted *p*‐values were calculated using the Benjamini–Hochberg correction method, and significantly different values are indicated by *p < 0.05 (*n* = 6). [Color figure can be viewed at wileyonlinelibrary.com]

Changes in assimilate allocation and root structure under salinity stress may result in whole‐plant metabolite alterations. An untargeted metabolomic approach was used to characterize the metabolic consequences of salinity stress in the leaves and roots of *H. vulgare*, *H. glaucum,* and *H. marinum* plants. This approach identified and determined the relative concentrations of 140 metabolites (Table S1), of which 132 showed differential accumulation (FDR < 0.05) in at least one species or one tissue, indicating extensive metabolic re‐composition under salinity stress. The first principal component (PC1) clearly revealed distinct accumulation patterns of leaf and root metabolites, while PC2 separated the samples of control and salinity stress conditions (Figure 3A). Overall, *H. vulgare* showed the strongest metabolic responses under salinity stress, reflecting a marked disruption of metabolic homoeostasis compared with less affected *H. glaucum* and *H. marinum*. The two salt‐tolerant species often exhibited similar patterns of metabolite accumulation under stress (Figures 3, S1; Table S1), indicating distinct strategies of adaptation to salinity.

Only four metabolites, porphobilinogen, mevalonate‐5P, Pro, and Val, were consistently accumulated under salinity in all three species (Table S1). Porphobilinogen is essential in plants for producing pigments like chlorophyll, which supports photosynthesis. It also contributes to haem production, influencing various metabolic and stress‐response pathways (Jaffe 2016). Mevalonate‐5P, as a pivotal intermediate in the mevalonate pathway, is a potential signal molecule that orchestrates plant growth and stress responses (Pu et al. 2021). A significant increase in Pro and Val contents indicates their universal role as osmoprotectants during abiotic stresses (Obata and Fernie 2012). In response to salinity, the 4‐oxo‐Pro and cis‐4‐hydroxy‐Pro levels were also strongly increased in *H. vulgare* and *H. glaucum* but dropped in *H. marinum* (Table S1).

In *H. vulgare* and *H. glaucum*, salinity stress induced a shift from high‐energy nucleotides (ATP, GDP and CTP) toward low‐energy nucleotides (AMP and GMP) in both shoots and roots (Figure 3B; Table S1), indicative of severe cellular energy depletion. This shift was less pronounced in *H. marinum*. The abundance of most glycolytic intermediates declined sharply in salt‐stressed tissues of all three species, with the strongest reductions observed in *H. vulgare* (Figure S1). Consistently, TCA cycle intermediates exhibited pronounced remodelling in both roots and shoots, again most evident in *H. vulgare* and minimal in *H. marinum* (Figure S1), likely reflecting species‐specific differences in metabolic resilience and maintenance of energy homoeostasis.

The levels of uridine derivatives (UTP, UDP, and UMP) were decreased under salinity stress conditions in all species and tissues (Figure 3B). In line with this observation, the levels of UDP‐Glc and UDP‐N‐acetylglucosamine, crucial intermediates of cell wall synthesis, were also strongly decreased in all species and tissues (Figure S1; Table S1). Furthermore, galacturonate‐1P, mannose‐6P, 6‐phosphogluconate, galactose‐1P, galacturonate, and glucuronate, all of which provide building blocks for cell wall synthesis, also showed decreased accumulation. These data indicate a massive reorganization of cell wall metabolism in response to salinity stress.

Regarding free amino acids, we observed a trend towards decreased levels in roots and higher levels in shoots in *H. vulgare* and *H. glaucum* (Figure 3C), with the exception of Pro and Val. *H. marinum* exhibited a distinct pattern of amino acid changes under salinity stress compared to the other species (Figure 3C).

To conclude, metabolite profiling indicated that *H. marinum* was least affected by salinity stress, followed by *H. glaucum*, whereas *H. vulgare* showed severe indications for energy deficiency and a stress‐associated metabolic imbalance in major pathways of central metabolism.

### 3.5 Species‐Specific Element Accumulation under Salinity Stress

Comparative analysis of Ca²⁺, Zn²⁺, Na⁺, K⁺, and Cl⁻ concentrations in roots and shoots of the three barley species revealed significant differences even under control conditions (Table 1), indicating species‐specific physiological requirements. Both salt‐tolerant species, *H. marinum* and *H. glaucum*, showed greater Ca^2+^ accumulation in their roots compared to *H. vulgare*. Among species, *H. marinum* roots accumulated the lowest Na^+^ but the highest K^+^ amounts. Also in the shoots, *H. marinum* exhibited enhanced levels of K^+^ and Zn^2+^, as compared to *H. vulgare* and *H. glaucum*.

**Table 1:** Element compositions in shoots and roots of *Hordeum vulgare*, *H. glaucum,* and *H. marinum* under control and salinity stress conditions.

	Control			Salinity		
Element	*H. vulgare*	*H. glaucum*	*H. marinum*	*H. vulgare*	*H. glaucum*	*H. marinum*
Shoots						
^44^Ca ^66^Zn ^23^Na ^39^K ^35^Cl	9.53 ± 0.50^a^ 9.73 ± 0.67^a^ 0.26 ± 0.78^a^ 69.30 ± 8.67^a^ n.d.	7.30 ± 0.62^b^ 8.24 ± 3.15^a^ 0.16 ± 0.04^b^ 63.11 ± 2.82^a^ n.d.	9.46 ± 0.76^a,b^ 19.61 ± 0.51^b^ 0.33 ± 0.05^a^ 85.41 ± 8.26^b^ n.d.	5.94 ± 1.35 ^A^ 18.19 ± 2.67 ^A^ 165.93 ± 28.82 ^A^ 42.74 ± 5.65 ^A^ 0.86 ± 0.39 ^A^	3.34 ± 0.69^B^ 16.95 ± 1.81 ^A^ 76.06 ± 24.79^B^ 32.12 ± 2.59^B^ 1.29 ± 0.08^B^	3.14 ± 0.37^B^ 16.81 ± 8.42 ^A^ 37.15 ± 6.35 ^C^ 37,28 ± 2.67 ^A,B^ 0.72 ± 0.04 ^C^
Roots						
^44^Ca ^66^Zn ^23^Na ^39^K ^35^Cl	5.74 ± 0.57^a^ 11.75 ± 2.66^a^ 5.66 ± 1.54^a^ 51.44 ± 6.25^a^ n.d.	13.32 ± 0.70^b^ 8.45 ± 0.76^a^ 3.30 ± 0.90^b^ 30.75 ± 2.70^b^ n.d.	8.15 ± 1.14^c^ 12.56 ± 1.18^a^ 1.59 ± 0.23^c^ 64.75 ± 3.10^c^ n.d.	2.40 ± 0.31 ^A^ 25.97 ± 0.00 ^A^ 117.93 ± 7.29 ^A^ 8.96 ± 0.44 ^A^ 1.28 ± 0.54 ^A^	1.86 ± 0.25^B^ 30.01 ± 3.00 ^A^ 106.33 ± 12.06 ^A^ 24.02 ± 1.66^B^ 0.84 ± 0.07^B^	1.68 ± 0.32^B^ 14.71 ± 0.93^B^ 85.71 ± 6.64^B^ 32.37 ± 4.63^C^ 0.73 ± 0.11^C^

*Note:* All values are shown in mg g^−1^ dry weight, with the exception of Zn, whose values are shown in µg g^−1^ dry weight. Significantly increased contents under salt stress in comparison to control conditions are highlighted in bold, significantly decreased in italic (Data are mean ± SD; *n* = 4–6, at *p* < 0.001 as determined by two‐tailed Student's *t*‐test between control and salinity stress conditions). Statistical significance among species was determined by one‐way ANOVA followed by Tukey's post hoc test (*p* < 0.05). Significant differences are indicated by different lowercase (a,b,c) and uppercase (A,B,C) letters for control and salinity conditions, respectively.

Salinity stress resulted in an increase in Na^+^, Cl^–^, and Zn^2+^ concentrations, accompanied by a significant decline in Ca^2+^ and K^+^ levels across all three species, although interspecific differences were evident (Table 1). *H. vulgare* accumulated the most Na⁺ and Cl⁻ ions in its roots, while *H. marinum* contained the lowest concentration. Regarding K^+^ retention, the species followed an opposite trend, with *H. marinum* maintaining the highest K^+^ content and *H. vulgare* the lowest. As a result, the Na^+^: K^+^ ratio in *H. marinum* roots was 2.7, compared to 4.4 in *H. glaucum* and as much as 13.2 in *H. vulgare*. These results indicate that *H. marinum* exhibits the strongest capacity to retain K⁺ and prevent its significant loss from the roots, thereby mitigating Na⁺‐induced cytotoxic effects.

We further compared ion distribution in the shoots versus roots under control and salt conditions. Data show that *H. vulgare* accumulated 1.4 times more Na^+^ in its shoots than in its roots, whereas *H. glaucum* accumulated 1.4 times less, and *H. marinum* even 2.3 times less Na^+^ in its shoots. The Na^+^: K^+^ ratio in salt‐stressed shoots was approximately 1.0 for *H. marinum*, 2.4 for *H. glaucum*, and 3.9 for *H. vulgare*. These results suggest that both wild barley species are capable of restricting Na^+^ transport to the shoots.

In contrast to Na^+^, Cl⁻ accumulated to high levels in both shoots and roots across all three barley species (Table 1), although *H. marinum* consistently maintained the lowest Cl⁻ concentrations.

**Figure 4:**
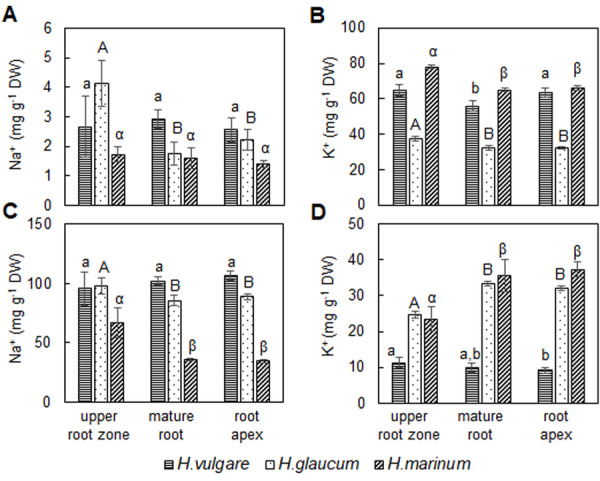
Na^+^ (A, C) and K^+^ (B, D) accumulation in different root zones of *H. vulgare*, *H. glaucum,* and *H. marinum* plants grown under control hydroponic (A, B) and salinity stress conditions (C, D). Data are mean ± SD; *n* = 4, one‐way ANOVA followed by a Tukey's HSD at *p* < 0.05, different letters denote significant differences between root zones values followed by the same letter (Latin lowercase for *H. vulgare*, Latin uppercase for *H. glaucum,* and Greek lowercase for *H. marinum*) do not differ significantly by ANOVA test at *p* < 0.05.

To investigate root zone‐specific accumulation of Na⁺ and K⁺ under salinity stress in more detail, we measured ion concentrations in the root tip and elongation zone (root apex), the mature root zone (mature root), and the upper part of the root system under both control and saline conditions (Figure 4). Under control conditions, low levels of Na⁺ were evenly distributed along the roots of both *H. vulgare* and *H. marinum* (Figure 4A) but not in *H. glaucum*, in which higher Na^+^ levels were detected in upper roots. Under salinity stress, *H. vulgare* exhibited a relatively uniform accumulation of very high Na⁺ levels across all root zones (Figure 4C). In contrast, *H. glaucum* showed preferential Na⁺ accumulation in the upper part of the root system compared with more distal root sections. A similar but more pronounced pattern was observed in *H. marinum*, where upper root parts accumulated approximately twice as much Na⁺ as the more distal root regions.

With respect to K⁺ accumulation, significantly higher concentrations were detected in the root tips and elongation zones of all three species under control conditions (Figure 4B). Salinity stress not only reduced overall K⁺ accumulation in the roots but also altered its spatial distribution in *H. glaucum* and *H. marinum*, where increased K⁺ levels were retained in the root tip and elongation zone as well as in the mature root zone, compared with the upper root zone. This altered distribution was not observed in *H. vulgare* (Figure 4D). Overall, *H. glaucum* and *H. marinum* retained higher K⁺ concentrations across all root sections than *H. vulgare* under salinity stress.

### 3.6 Na^+^ and K^+^ are Differently Distributed in the Root Tissues of Three Barley Species Under Salinity Stress

**Figure 5:**
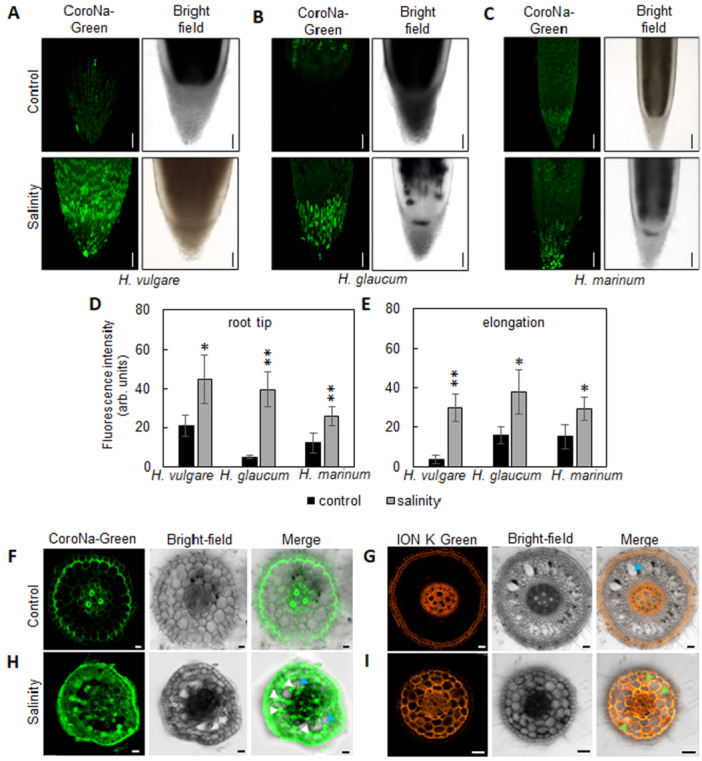
Accumulation and patterning profiles of Na^+^ and K^+^ in roots of barley species grown under control and salinity stress conditions. (A–C) The representative images of Na^+^ distribution within control (above left) and stressed roots (below left) of *H. vulgare* (A), *H. glaucum* (B), and *H. marinum* as visualized by CoroNa Green (C). Panels next to fluorescence images represent the respective bright field images. Bars, 100 µm. (D, E) Quantification of intensity of CoroNa Green fluorescence in root tips (D) and elongation zone (E). Data are mean ± SD; *n* = 3–4, *t* significant (Student's *t*‐test) at: *p < 0.05, **p < 0.01. (F–I) Na^+^ (F, H) and K^+^ distribution (G, I) in the upper parts of *H. marinum* roots grown under control (F, G) and salinity stress conditions (H, I), as analysed by CoroNa Green and ION Potassium Green‐2 AM staining, correspondingly. Cells filled by Na^+^ are indicated by white arrowheads, K^+^ ‐ by green arrowheads, and the aerenchyma zone ‐ by blue arrowheads. Bars, 50 µm. [Color figure can be viewed at wileyonlinelibrary.com]

To get more insights into tissue‐specific Na⁺ and K⁺ distribution within roots under salinity stress, we first examined Na⁺ localization in the roots of the three Hordeum species using CoroNa Green staining. This analysis revealed that all three species accumulated high levels of Na⁺ in the root cap and elongation zone; however, Na⁺ accumulation was most pronounced in salt‐stressed *H. vulgare*, followed by *H. glaucum* and *H. marinum* (Figure 5A‐E).

Next, we analysed the cellular distribution of Na⁺ and K⁺. In the upper root regions of *H. marinum*, where lateral and adventitious root branching is initiated, and aerenchyma development is prominent, using ion‐specific fluorescent dyes CoroNa Green for Na⁺ and ION Potassium Green for K⁺, respectively. Both dyes freely diffuse across cell membranes and enter the cells, where they are cleaved by intracellular esterases to generate their ion‐responsive forms (Fujishima et al. 2021). Prior to localization analyses, we confirmed differences in the cell‐type‐specific distribution of ION Potassium Green‐2 AM for K⁺ relative to CoroNa Green AM for Na⁺ in salt‐stressed roots (Figure S2). Unlike CoroNa Green, ION Potassium Green was readily detectable even in bright‐field mode. Both dyes were subsequently applied to cross‐sections taken 10 mm below the root collar to visualize Na⁺ and K⁺ distribution (Figure 5F‐I). In control roots, Na⁺ signals were detected in the thin cytosol layer of epidermal and subepidermal cells, as well as in the stele surrounding the vascular bundles (Figure 5F). The distribution of K⁺ in control roots was largely similar to that of Na⁺ (Figure 5G). In salt‐treated *H. marinum* roots, Na⁺ signals were observed in the most cell types and, additionally, showed strong enrichment presumably in the vacuoles of numerous cortical cells (Figure 5H). ION Potassium Green fluorescence was also visible in the cytosol of cells throughout the root tissues. In contrast to *H. marinum*, the upper root regions of the other barley species did not exhibit pronounced changes in cellular ion distribution or significant enrichment of Na⁺‐accumulating cells under salinity stress (Figure S3).

To conclude, a comparative analysis of cultivated varieties and wild barley species revealed that salinity resistance increased from *H. vulgare* to *H. glaucum* to *H. marinum*. While H. vulgare was most severely affected, *H. glaucum* maintained tissue water content and root assimilate allocation despite reduced growth and carbon fixation. *H. marinum* showed the highest tolerance under 300 mM NaCl, outperforming the other species.

## 4 Discussion

Salinity and waterlogging severely impair the plant growth of various barley cultivars, yet some wild species have evolved effective mechanisms to survive and thrive under detrimental conditions. Here, we discover a novel root‐centred adaptive strategy that enables *H. marinum* to thrive under salinity in stagnant hydroponic conditions, integrating anatomical plasticity, metabolic efficiency, and ion control.

All three barley species, *H. vulgare*, *H. glaucum,* and *H. marinum*, accumulated proline when grown under stagnant hydroponic conditions supplemented with 300 mM NaCl, consistent with its role as a key adaptive metabolite under both salinity stress (Arias‐Baldrich et al. 2015) and waterlogging (Savchenko et al. 2019). Unlike the others, *H. marinum* additionally accumulated several amino acids (Figure 2, Table S1) that may function as osmoprotectants. It also showed elevated levels of TCA cycle intermediates (citrate, 2‐oxoglutarate, and succinate), suggesting maintenance of metabolic activity under salinity. In contrast, *H. vulgare* displayed signs of metabolic strain, with increased accumulation of osmolytes and stress‐related compounds (sugar alcohols, allantoin, polyamines, taurine, and haem metabolites (Table S1), indicative of higher metabolic costs during stress.

Abiotic stress in plants is frequently associated with the accumulation of ureide compounds (Irani and Todd 2016; Kaur et al. 2023). Exogenous application of allantoin has been shown to reduce reactive oxygen species‐induced K⁺ loss from barley roots, highlighting its protective role in maintaining K⁺ homoeostasis during salinity or oxidative stress (Shabala et al. 2016). Ureide accumulation patterns differed among the barley species. While *H. vulgare* and *H. glaucum* accumulated elevated allantoin levels under salinity stress, *H. marinum* maintained consistently higher ureide levels. This constitutively elevated ureide pool in *H. marinum* may represent a species‐specific adaptation that enhances stress resilience and supports K⁺ retention.

**Figure 6:**
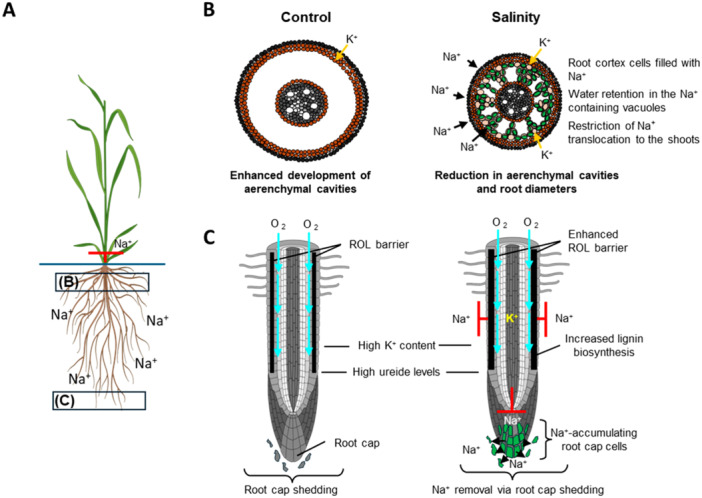
Schematic overview of the main processes occurring during incubation under stagnant hydroponic conditions (control) and salinity stress in different root zones of *H. marinum*. (A) Experimental design. Root regions shown in detail in (B) and (C) are indicated by black boxes. (B, C) Adaptive processes occurring in the upper parts of newly developed adventitious and lateral roots (B), and in the root tips and elongation zones (C) of *H. marinum* in response to stagnant hydroponic waterlogging (left) and combined salinity and stagnant hydroponic waterlogging stress (right). Black arrows indicate Na⁺ transport, blue arrows indicate O₂ movement, and red T‐bars indicate restriction of Na⁺ transport. Cells accumulating Na⁺, presumably in vacuoles, are shown in green; the cytosol, with K⁺ likely retained, is shown in orange; and green patches indicate intracellular Na⁺ distribution. Abbreviation: ROL, radial oxygen loss. In addition to the current results, the scheme includes related scientific data from (Malik et al. 2008; Xu et al. 2022; Isayenkov et al. 2020). Images were created with partial support from BioRender. (https://biorender.com **)**. [Color figure can be viewed at wileyonlinelibrary.com]

Excessive Na⁺ and Cl⁻ accumulation is a primary driver of salinity‐induced damage. *H. marinum* accumulated the least Na⁺ overall and, together with *H. glaucum*, restricted Na⁺ translocation to shoots, retaining it mainly in roots (Figure 6; Table 1). In contrast, *H. vulgare* exhibited a Na⁺ shoot‐includer phenotype, accumulating high Na⁺ levels in aerial tissues and showing pronounced root K⁺ loss under stress (Figure S4), which further aggravated ionic imbalance. Beyond Na⁺ exclusion, *H. marinum* also more tightly regulates Cl⁻ distribution, indicating superior control of ion transport and tissue partitioning.

Fluorescence imaging showed that Na⁺ accumulated predominantly in the root apex of all three barley species, consistent with observations in salt‐tolerant wheat (Wu et al. 2015). The root apex is considered a key site for signal perception and integration (Baluška et al. 2010) and has been proposed to function as a salt sensor (Wu et al. 2015). The preferential accumulation of Na⁺ in this region may represent a broader strategy to restrict ion transport to sensitive tissues. Because root apex cells are continuously shed during development (Kumpf and Nowack 2015), their turnover could facilitate the removal of excess Na⁺. Under stress conditions, enhanced turnover of Na^+‐^accumulating cells may therefore serve as a protective mechanism, enabling the removal of excess Na⁺ while safeguarding the meristematic zone (Figures 6 and S4). Localization of the Na⁺/H⁺ antiporter SOS1 to the root apex (Shi et al. 2002) further supports a role for this region in Na⁺ detoxification.

Na⁺ accumulation in the elongation and mature root zones was moderate in the two salt‐tolerant barley species compared with *H. vulgare*, indicating the presence of effective barriers that limit Na^+^ entry. These barriers may involve enhanced cell wall thickening and modification in the exodermis (Dabravolski and Isayenkov 2023; Tariq et al. 2026). Strengthening apoplastic barriers may simultaneously reduce radial oxygen loss, thereby mitigating hypoxia stress. In this way, salt‐tolerant Hordeum species may simultaneously limit ion entry and improve internal oxygen retention (Figures 6 and S4b).

While salinity stress inhibited the formation of lateral and adventitious roots in *H. vulgare*, the two halophytic barley species developed a large number of new roots (Figures 1G,H and 2A–G), which, however, exhibited substantial anatomical modifications. In *H. marinum*, salinity likely did not induce extensive programmed cell death–mediated cortical cell loss during aerenchyma formation. Instead, cortical cells were largely retained and likely contributed to sequestration of excess Na⁺, most probably into vacuoles (Figure 5H). Given that vacuoles can occupy up to 90% of the cell volume (Isayenkov et al. 2010), this strategy would mitigate salinity‐induced dehydration (Figure 6). Concurrent cytosolic K⁺ retention and upregulation of the non‐symbiotic haemoglobin 1 gene (Isayenkov et al. 2020) suggest coordinated mechanisms supporting ionic balance and oxygen buffering under stress.

By including *H. glaucum* as an intermediate genotype and directly comparing it with both the halophytic *H. marinum* and the cultivated *H. vulgare*, this study resolves gradients of adaptive traits rather than binary differences.

In conclusion, stress tolerance in *H. marinum* arises from the coordinated integration of root‐centred metabolic stability, ion partitioning, anatomical plasticity, and oxygen buffering, rather than from any single protective mechanism. However, the key advance of this work is the demonstration that these traits operate as an integrated system and vary quantitatively across Hordeum species. This comparative, multi‐level perspective highlights the root system as the central hub of adaptive regulation and provides a more mechanistic framework for identifying transferable traits for crop improvement. Remaining questions regarding Na⁺ storage capacity in newly formed roots, additional exclusion mechanisms, and Cl⁻ regulation represent important directions for future research.

## Conflicts of Interest

The authors declare no conflicts of interest.

## Supporting information

Supporting File 1

Supporting Movie 2

Supporting Movie 3

Supporting File 4

Supporting File 5

## Data Availability

The data that support the findings of this study are available on request from the corresponding author. The data are not publicly available due to privacy or ethical restrictions.

## References

[pce70563-bib-0001] Alamri, S. A. , E. G. Barrett‐Lennard , N. L. Teakle , and T. D. Colmer . 2013. “Improvement of Salt and Waterlogging Tolerance in Wheat: Comparative Physiology of *Hordeum marinum*‐*Triticum aestivum* Amphiploids With Their *H. marinum* and Wheat Parents.” Functional Plant Biology 40: 1168–1178. 32481184 10.1071/FP12385

[pce70563-bib-0002] Arias‐Baldrich, C. , N. Bosch , D. Begines , A. B. Feria , J. A. Monreal , and S. García‐Mauriño . 2015. “Proline Synthesis in Barley under Iron Deficiency and Salinity.” Journal of Plant Physiology 183: 121–129. 26125122 10.1016/j.jplph.2015.05.016

[pce70563-bib-0003] Baluška, F. , S. Mancuso , D. Volkmann , and P. W. Barlow . 2010. “Root Apex Transition Zone: A Signalling‐Response Nexus in the Root.” Trends in Plant Science 15: 402–408. 20621671 10.1016/j.tplants.2010.04.007

[pce70563-bib-0004] Barrett‐Lennard, E. G. 2003. “The Interaction Between Waterlogging and Salinity in Higher Plants: Causes, Consequences and Implications.” Plant and Soil 253: 35–54.

[pce70563-bib-0005] Borisjuk, L. , P. Horn , K. Chapman , P. M. Jakob , A. Gündel , and H. Rolletschek . 2023. “Seeing Plants as Never Before.” New Phytologist 238: 1775–1794. 36895109 10.1111/nph.18871

[pce70563-bib-0006] Chen, M. , H. Gao , and Y. Tu , et al. 2025. “Transcriptomic and Metabolomic Analysis Uncover Core Salt‐Responsive Elements in *Hordeum marinum* .” Plant Physiology and Biochemistry 229: 110618. 41398755 10.1016/j.plaphy.2025.110618

[pce70563-bib-0007] Colmer, T. D. , T. J. Flowers , and R. Munns . 2006. “Use of Wild Relatives to Improve Salt Tolerance in Wheat.” Journal of Experimental Botany 57: 1059–1078. 16513812 10.1093/jxb/erj124

[pce70563-bib-0008] Dabravolski, S. A. , and S. V. Isayenkov . 2023. “The Regulation of Plant Cell Wall Organisation under Salt Stress.” Frontiers in Plant Science 14: 1118313. 36968390 10.3389/fpls.2023.1118313 PMC10036381

[pce70563-bib-0009] Evans, D. E. 2004. “Aerenchyma Formation.” New Phytologist 161: 35–49.

[pce70563-bib-0010] Feng, H. , Q. Du , Y. Jiang , et al. 2025. “Hordeum I Genome Unlocks Adaptive Evolution and Genetic Potential for Crop Improvement.” Nature Plants 11: 438–452. 40087544 10.1038/s41477-025-01942-w PMC11928320

[pce70563-bib-0011] Food and Agriculture Organization . 2016. “The State of Food and Agriculture: Climate Change, Agriculture and Food Security.” Roma, Italy, p. 173.

[pce70563-bib-0012] Fujishima, A. , K. Takahashi , and M. Goto , et al. 2021. “Live Visualisation of Electrolytes During Mouse Embryonic Development Using Electrolyte Indicators.” PLoS One 16: e0246337. 33513193 10.1371/journal.pone.0246337 PMC7845971

[pce70563-bib-0013] Gao, H. , M. Chen , and N. Jin , et al. 2025. “A Comprehensive Analytical Method ‘Regulatome’ Revealed a Novel Pathway for Aerenchyma Formation under Waterlogging in Wheat.” Physiologia Plantarum 177: e70157. 40083176 10.1111/ppl.70157

[pce70563-bib-0014] Garthwaite, A. J. , R. Bothmer , and T. D. Colmer . 2003. “Diversity in Root Aeration Traits Associated With Waterlogging Tolerance in the Genus Hordeum.” Functional Plant Biology 30: 875–889. 32689072 10.1071/FP03058

[pce70563-bib-0015] Garthwaite, A. J. , R. von Bothmer , and T. D. Colmer . 2005. “Salt Tolerance in Wild Hordeum Species Is Associated With Restricted Entry of Na^+^ and Cl– into the Shoots.” Journal of Experimental Botany 56: 2365–2378. 16014366 10.1093/jxb/eri229

[pce70563-bib-0016] Herzog, M. , E. Pellegrini , and O. Pedersen . 2023. “A Meta‐Analysis of Plant Tissue O_2_ Dynamics.” Functional Plant Biology 50: 519–531. 37160400 10.1071/FP22294

[pce70563-bib-0017] Huang, L. , L. Kuang , X. Li , L. Wu , D. Wu , and G. Zhang . 2018. “Metabolomic and Transcriptomic Analyses Reveal the Reasons Why *Hordeum marinum* Has Higher Salt Tolerance Than *Hordeum vulgare* .” Environmental and Experimental Botany 156: 48–61.

[pce70563-bib-0018] Irani, S. , and C. D. Todd . 2016. “Ureide Metabolism under Abiotic Stress in *Arabidopsis thaliana* .” Journal of Plant Physiology 199: 87–95. 27302009 10.1016/j.jplph.2016.05.011

[pce70563-bib-0019] Isayenkov, S. , A. Hilo , P. Rizzo , et al. 2020. “Adaptation Strategies of Halophytic Barley *Hordeum marinum* ssp. *marinum* to High Salinity and Osmotic Stress.” International Journal of Molecular Sciences 21: 9019. 33260985 10.3390/ijms21239019 PMC7730945

[pce70563-bib-0020] Isayenkov, S. , J. C. Isner , and F. J. M. Maathuis . 2010. “Vacuolar Ion Channels: Roles in Plant Nutrition and Signalling.” FEBS Letters 584: 1982–1988. 20188732 10.1016/j.febslet.2010.02.050

[pce70563-bib-0021] Isayenkov, S. V. 2019. “Genetic Sources for the Development of Salt Tolerance in Crops.” Plant Growth Regulation 89: 1–17.

[pce70563-bib-0022] Isayenkov, S. V. 2023. “Wild Barley Relatives ‐ Potential Donors of Salinity Tolerance for Cereal Crops.” In Multiple abiotic stress tolerance in higher plants: Addressing the growing challenges, edited by N. K. Gupta , Yu Shavrukov , and R. Singhal . CRC Press.

[pce70563-bib-0023] Islam, S. , A. Malik , A. Islam , and T. Colmer . 2007. “Salt Tolerance in a *Hordeum marinum*‐*Triticum aestivum* Amphiploid, and Its Parents.” Journal of Experimental Botany 58: 1219–1229. 17283374 10.1093/jxb/erl293

[pce70563-bib-0024] Jaffe, E. K. 2016. “The Remarkable Character of Porphobilinogen Synthase.” Accounts of Chemical Research 49: 2509–2517. 27783504 10.1021/acs.accounts.6b00414 PMC5148690

[pce70563-bib-0025] Jin, N. , Z. Cai , L. Ye , Q. Shen , G. Zhang , and Z. Xu . 2025. “Improvement of Waterlogging Tolerance in Wheat by the Stress Priming Through Inducing Aerenchyma Formation.” Plant Growth Regulation 105: 245–255.

[pce70563-bib-0026] Kaur, R. , J. Chandra , B. Varghese , and S. Keshavkant . 2023. “Allantoin: A Potential Compound for the Mitigation of Adverse Effects of Abiotic Stresses in Plants.” Plants 12: 3059. 37687306 10.3390/plants12173059 PMC10489999

[pce70563-bib-0027] Konnerup, D. , A. I. Malik , A. K. M. R. Islam , and T. D. Colmer . 2017. “Evaluation of Root Porosity and Radial Oxygen Loss of Disomic Addition Lines of *Hordeum Marinum* in Wheat.” Functional Plant Biology 44: 400–409. 32480573 10.1071/FP16272

[pce70563-bib-0028] Kotula, L. , P. L. Clode , G. G. Striker , et al. 2015. “Oxygen Deficiency and Salinity Affect Cell‐Specific Ion Concentrations in Adventitious Roots of Barley (*Hordeum vulgare*).” New Phytologist 208: 1114–1125. 26094736 10.1111/nph.13535

[pce70563-bib-0029] Kuang, L. , Q. Shen , and L. Chen , et al. 2022. “The Genome and Gene Editing System of Sea Barley Grass Provide a Novel Platform for Cereal Domestication and Stress Tolerance Studies.” Plant Communications 3: 100333. 35643085 10.1016/j.xplc.2022.100333 PMC9482977

[pce70563-bib-0030] Kumpf, R. P. , and M. K. Nowack . 2015. “The Root Cap: A Short Story of Life and Death.” Journal of Experimental Botany 66: 5651–5662. 26068468 10.1093/jxb/erv295

[pce70563-bib-0031] Lee, H. B. , D. H. Jeong , and J. S. Park . 2022. “Accumulation Patterns of Intracellular Salts in a New Halophilic Amoeboflagellate, *Euplaesiobystra salpumilio* sp. nov., (Heterolobosea; Discoba) under Hypersaline Conditions.” Frontiers in Microbiology 13: 960621. 35992684 10.3389/fmicb.2022.960621 PMC9389213

[pce70563-bib-0032] Lindermayr, C. , and J. Durner . 2015. “Interplay of Reactive Oxygen Species and Nitric Oxide: Nitric Oxide Coordinates Reactive Oxygen Species Homeostasis.” Plant Physiology 167: 1209–1210. 25819986 10.1104/pp.15.00293 PMC4378185

[pce70563-bib-0033] Liu, K. , M. T. Harrison , and A. Ibrahim , et al. 2020. “Genetic Factors Increasing Barley Grain Yields under Soil Waterlogging.” Food and Energy Security 9: e238.

[pce70563-bib-0034] Luan, H. , B. Guo , Y. Pan , C. Lv , H. Shen , and R. Xu . 2018. “Morpho‐Anatomical and Physiological Responses to Waterlogging Stress in Different Barley (*Hordeum vulgare* L.) Genotypes.” Plant Growth Regulation 85: 399–409.

[pce70563-bib-0035] Malik, A. I. , J. P. English , and T. D. Colmer . 2009. “Tolerance of *Hordeum marinum* Accessions to O_2_ Deficiency, Salinity and These Stresses Combined.” Annals of Botany 103: 237–248. 18701600 10.1093/aob/mcn142 PMC2707305

[pce70563-bib-0036] Manik, N. S. M. , M. D. Quamruzzaman , and M. Livermore , et al. 2022a. “Impacts of Barley Root Cortical Aerenchyma on Growth, Physiology, Yield Components, and Grain Quality under Field Waterlogging Conditions.” Field Crops Research 279: 108461.

[pce70563-bib-0037] Manik, S. M. N. , M. Quamruzzaman , C. Zhao , et al. 2022b. “Genome‐Wide Association Study Reveals Marker Trait Associations (Mta) for Waterlogging‐Triggered Adventitious Roots and Aerenchyma Formation in Barley.” International Journal of Molecular Sciences 23: 3341. 35328762 10.3390/ijms23063341 PMC8954902

[pce70563-bib-0038] Maršálová, L. , P. Vítámvás , R. Hynek , I. T. Prášil , and K. Kosová . 2016. “Proteomic Response of *Hordeum vulgare* cv. Tadmor and *Hordeum Marinum* to Salinity Stress: Similarities and Differences Between a Glycophyte and a Halophyte.” Frontiers in Plant Science 7: 1154. 27536311 10.3389/fpls.2016.01154 PMC4971088

[pce70563-bib-0040] Munns, R. , and M. Gilliham . 2015. “Salinity Tolerance of Crops—What Is the Cost?” New Phytologist 208: 668–673. 26108441 10.1111/nph.13519

[pce70563-bib-0041] Munz, E. , H. Rolletschek , S. Oeltze‐Jafra , et al. 2017. “A Functional Imaging Study of Germinating Oilseed Rapeseed.” New Phytologist 216: 1181–1190. 28800167 10.1111/nph.14736

[pce70563-bib-0042] Obata, T. , and A. R. Fernie . 2012. “The Use of Metabolomics to Dissect Plant Responses to Abiotic Stresses.” Cellular and Molecular Life Sciences 69: 3225–3243. 22885821 10.1007/s00018-012-1091-5 PMC3437017

[pce70563-bib-0043] Palmgren, M. , and S. Shabala . 2024. “Adapting Crops for Climate Change: Regaining Lost Abiotic Stress Tolerance in Crops.” Frontiers in Science 2: 1416023.

[pce70563-bib-0044] Patel, S. 2016. “Salicornia: Evaluating the Halophytic Extremophile as a Food and a Pharmaceutical Candidate.” 3 Biotech 6: 104. 10.1007/s13205-016-0418-6 PMC4835422 28330174

[pce70563-bib-0045] Pu, X. , X. Dong , Q. Li , Z. Chen , and L. Liu . 2021. “An Update on the Function and Regulation of Methylerythritol Phosphate and Mevalonate Pathways and Their Evolutionary Dynamics.” Journal of Integrative Plant Biology 63: 1211–1226. 33538411 10.1111/jipb.13076

[pce70563-bib-0046] Rolletschek, H. , L. Borisjuk , E. M. Gómez‐Álvarez , and C. Pucciariello . 2024. “Advances in Seed Hypoxia Research.” Plant Physiology 197: kiae556. 39471319 10.1093/plphys/kiae556 PMC11852284

[pce70563-bib-0047] Savchenko, T. , H. Rolletschek , N. Heinzel , K. Tikhonov , and K. Dehesh . 2019. “Waterlogging Tolerance Rendered by Oxylipin‐Mediated Metabolic Reprogramming in Arabidopsis.” Journal of Experimental Botany 70: 2919–2932. 30854562 10.1093/jxb/erz110 PMC6506769

[pce70563-bib-0048] Shabala, L. , J. Zhang , I. Pottosin , et al. 2016. “Cell‐Type‐Specific H^+^‐ATPase Activity in Root Tissues Enables K^+^ Retention and Mediates Acclimation of Barley (*Hordeum vulgare*) to Salinity Stress.” Plant Physiology 172: 2445–2458. 27770060 10.1104/pp.16.01347 PMC5129721

[pce70563-bib-0049] Sharmita, O. , A. B. Siddique , and K. Liu , et al. 2026. “Boosting Crop Resilience to Waterlogging Through Hormone‐Regulated Root Traits.” European Journal of Agronomy 174: 127948.

[pce70563-bib-0050] Shi, H. , F. J. Quintero , J. M. Pardo , and J. K. Zhu . 2002. “The Putative Plasma Membrane Na^+^/H^+^ Antiporter SOS1 Controls Long‐Distance Na^+^ Transport in Plants.” Plant Cell 14: 465–477. 11884687 10.1105/tpc.010371 PMC152925

[pce70563-bib-0051] Tariq, F. , L. Zhao , and S. Hussain , et al. 2026. “Plasticity and Adaptive Architecture of Roots for Enhanced Salinity Tolerance in Crops.” Biotechnology Advances 87: 108773. 41344580 10.1016/j.biotechadv.2025.108773

[pce70563-bib-0053] Wu, H. , L. Shabala , and X. Liu , et al. 2015. “Linking Salinity Stress Tolerance With Tissue‐Specific Na^+^ Sequestration in Wheat Roots.” Frontiers in Plant Science 6: 71. 25750644 10.3389/fpls.2015.00071 PMC4335180

[pce70563-bib-0054] Xu, Z. , Q. Shen , and G. Zhang . 2022. “The Mechanisms for the Difference in Waterlogging Tolerance Among Sea Barley, Wheat and Barley.” Plant Growth Regulation 96: 431–441.

[pce70563-bib-0055] Xu, Z. , Z. Wang , Y. Zheng , H. Gao , Q. Shen , and G. Zhang . 2025. “Sea Barley: Evolutionary Insights and Potential for Crop Improvement.” *Journal of Integrative Agriculture (In Press)*. 10.1016/j.jia.2025.10.010..

[pce70563-bib-0056] Yamauchi, T. , T. D. Colmer , O. Pedersen , and M. Nakazono . 2018. “Regulation of Root Traits for Internal Aeration and Tolerance to Soil Waterlogging‐Flooding Stress.” Plant Physiology 176: 1118–1130. 29118247 10.1104/pp.17.01157 PMC5812745

[pce70563-bib-0057] Zhang, X. , Y. Fan , S. Shabala , et al. 2017. “A New Major‐Effect QTL for Waterlogging Tolerance in Wild Barley (*H. spontaneum*).” Theoretical and Applied Genetics 130: 1559–1568. 28447117 10.1007/s00122-017-2910-8

[pce70563-bib-0058] Zhang, Y. , X. Wang , and M. M. Malko , et al. 2025. “The Physiological Mechanisms of Waterlogging Priming on Aerenchyma Formation in Secondary Roots of Wheat Under Waterlogging Stress.” Environmental and Experimental Botany 237: 106207.

