# Supplementary material for: Root Structural and Metabolic Plasticity Confers Tolerance to Salinity in Wild Barley Species Grown Under Waterlogging
Source: Plant Cell Environ. 2026 Apr 23;49(8):5593–606. doi: 10.1111/pce.70563 (PMC13353622; doi:10.1111/pce.70563)
Supplement: Supplementary file 1 — Supporting File 1 [file PCE-49-5593-s003.pdf]

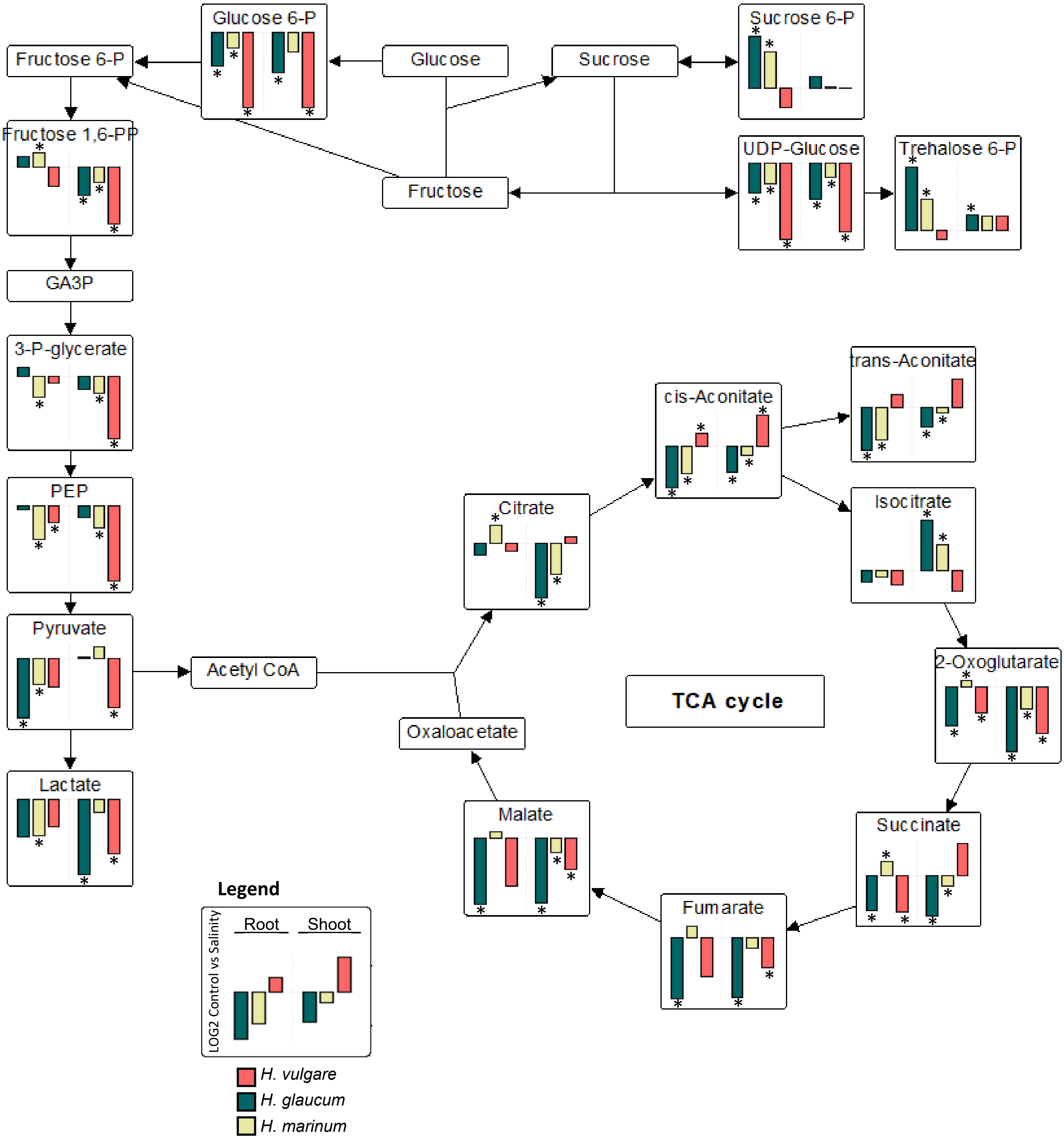

Figure S1. Changes in content of metabolites involved in glycolysis and TCA cycle in roots and shoots of *H. vulgare*, *H. glaucum* and *H. marinum* plants grown under control and salinity stress conditions. Data are shown as LOG_2 values of the relation salinity to control, negative values mean decreased metabolite content, positive values – increased metabolite content. Adjusted p-values were calculated using Benjamini-Hochberg correction and significantly different values are indicated by * at $P < 0.05$ (n = 6).

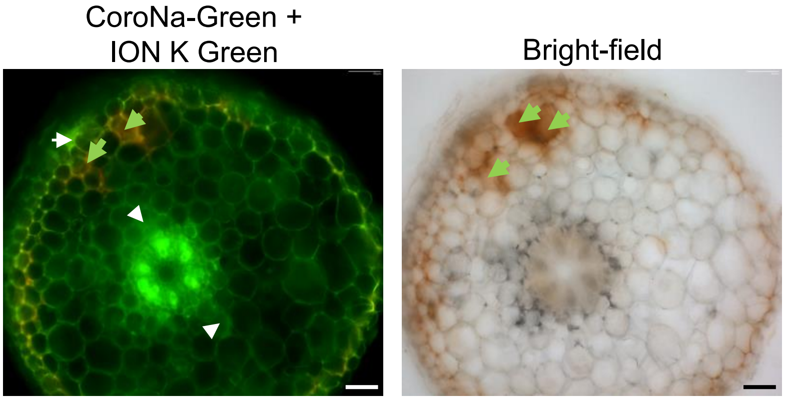

Figure S2. Distribution of K^+-specific ION Potassium Green and Na^+-specific CoroNa Green AM stains in salt-stressed *H. marinum* root. Na^+ labeled by CoroNa Green AM is indicated by white arrowheads, K^+ labelled by ION Potassium Green - by green arrowheads. Bars, 50 µm.

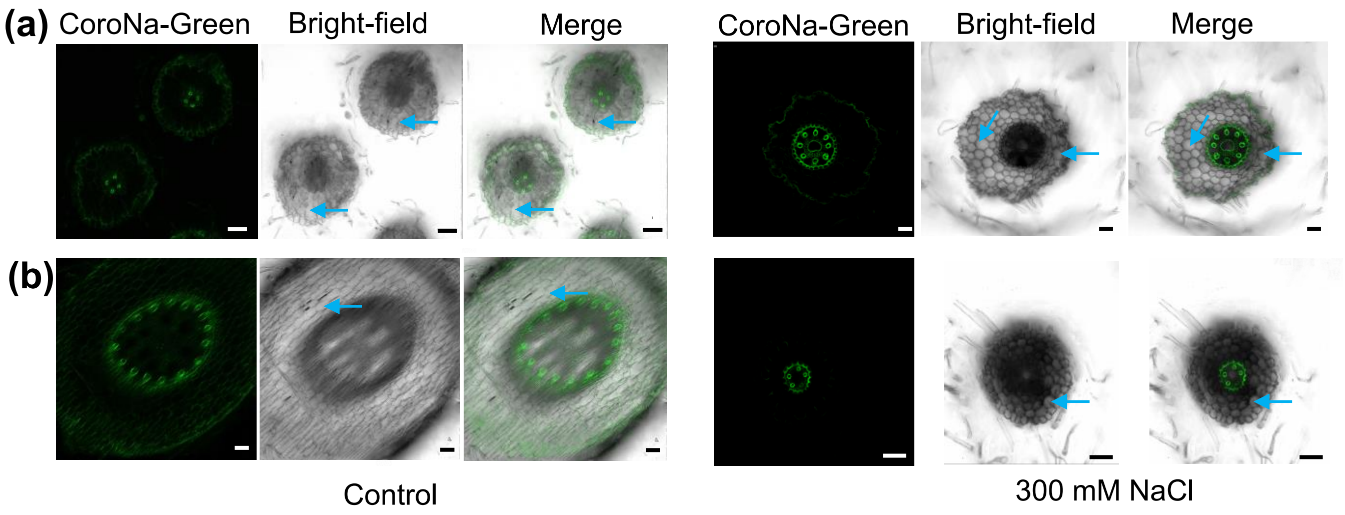

Figure S3. Na^{+} distribution in the upper parts of H. vulgare (a) and H. glaucum (b) roots grown under control (left panel) and salinity stress (right panel) conditions. Aerenchyma presence is indicated by blue arrowheads. Bars, 50 µm.

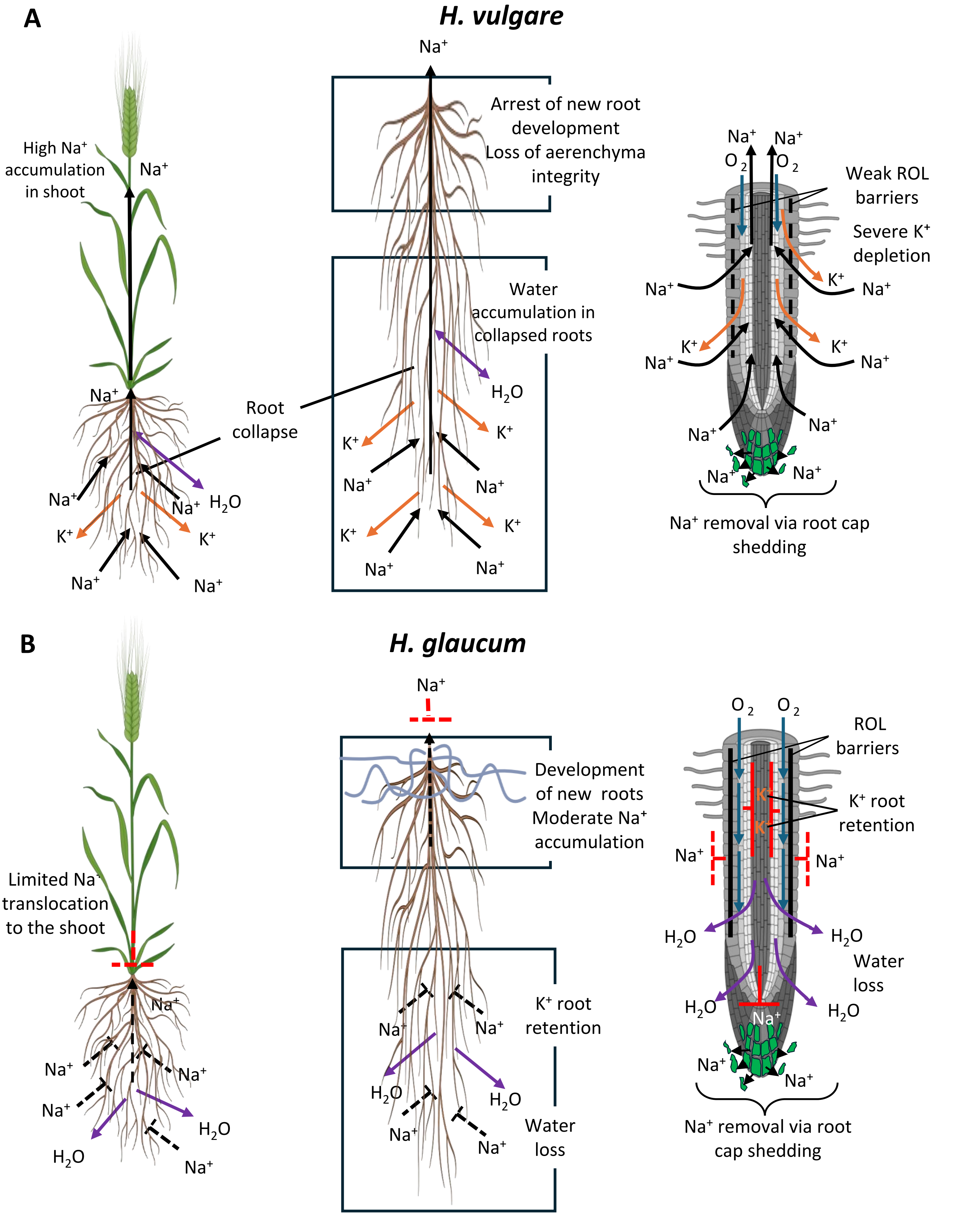

Figure S4. A schematic overview of the main processes occurring during salinity stress in stagnant hydroponic conditions in *H. vul*gare (A) and *H. glaucum* (B) at the plant level (left), across different root zones (middle) and within a root (right). Black arrows indicate the directions of Na^+ movement, orange arrows indicate movement of K+, blue arrows reflect the movement of O2, purple arrows highlight the water movement, and green patches indicate Na^+ within cells. Abbreviation: ROL, radial oxygen loss. (Created in https://BioRender.com).
